# ‘I know those people will be approachable and not mistreat us’: a qualitative study of inspectors and private drug sellers’ views on peer supervision in rural Uganda

**DOI:** 10.1186/s12992-020-00636-6

**Published:** 2020-10-27

**Authors:** Arthur Bagonza, Stefan Peterson, Andreas Mårtensson, Milton Mutto, Phyllis Awor, Freddy Kitutu, Linda Gibson, Henry Wamani

**Affiliations:** 1 Department of Community Health and Behavioural Sciences, Makerere University College of Health Sciences, School of Public Health, Kampala, Uganda; 2 Department of Health Policy Planning and Management, Makerere University College of Health Sciences, School of Public Health, Kampala, Uganda; 3 Department of Women’s and Children’s Health, International Maternal and Child Health Unit, Uppsala University, Uppsala, Sweden; 4 Department of Disease Control and Environmental Health, Makerere University College of Health Sciences, School of Public Health, Kampala, Uganda; 5 Department of Pharmacy, School of Health Sciences, Makerere University College of Health Sciences, Kampala, Uganda; 6 School of Social Sciences, Nottingham Trent University, Nottingham, UK

**Keywords:** Drug sellers, Inspection, Pharmaceutical sector, peer supervision, Grounded theory, Uganda

## Abstract

**Background:**

Peer supervision improves health care delivery by health workers. However, in rural Uganda, self-supervision is what is prescribed for licensed private drug sellers by statutory guidelines. Evidence shows that self-supervision encourages inappropriate treatment of children less than 5 years of age by private drug sellers. This study constructed a model for an appropriate peer supervisor to augment the self-supervision currently practiced by drug sellers at district level in rural Uganda.

**Methods:**

In this qualitative study, six Key informant interviews were held with inspectors while ten focus group discussions were conducted with 130 drug sellers. Data analysis was informed by the Kathy Charmaz constructive approach to grounded theory. Atlas ti.7 software package was used for data management.

**Results:**

A model with four dimensions defining an appropriate peer supervisor was developed. The dimensions included; incentives, clearly defined roles, mediation and role model peer supervisor. While all dimensions were regarded as being important, all participants interviewed agreed that incentives for peer supervisors were the most crucial. Overall, an appropriate peer supervisor was described as being exemplary to other drug sellers, operated within a defined framework, well facilitated to do their role and a good go-between drug sellers and government inspectors.

**Conclusion:**

Four central contributions advance literature by the model developed by our study. First, the model fills a supervision gap for rural private drug sellers. Second, it highlights the need for terms of reference for peer supervisors. Third, it describes who an appropriate peer supervisor should be. Lastly, it elucidates the kind of resources needed for peer supervision.

## Background

Globally, effectiveness of health systems has been in part, associated with adequate clinical supervision of human resources for health [1]. Different scholars suggest that effective support supervision in the delivery of basic health services may be defined as a way of guiding, helping and teaching health providers in a manner that fosters two-way communication between supervisors and supervisees at the work place [2, 3]. Besides communication being two-way, many supervisees feel that support supervision has had an effect on their work when there is constructive feedback [4–6].

In sub-Saharan Africa, Uganda inclusive, while supervision of public health facilities is clearly defined [2], supervision of rural private health care providers predominantly comprised of drug shops is left to licensed drug sellers whose ultimate objective is to make profit [7]. This has been associated with unsatisfactory quality of care offered to patients in general and in some contexts, has been linked to inappropriate treatment of febrile children less than 5 years by drug sellers [8, 9]. In other instances, self-supervision which is characterised by drug sellers not consulting others a lot if at all they do, has been associated with charging high prices for drugs since dispensing of drugs is based on the patient’s choice [10]. One cardinal challenge with self-supervision is that its effectiveness has not been documented and therefore may be unsuitable to adopt in a dynamic sub-sector such as one involving drug sellers in Uganda.

As such, different models of supervision have been developed over time [11]. However, whereas earlier models of supervision targeted the supervisee as a recipient of knowledge and instruction from the supervisor, later models recognised the need to include supervisors and institutions charged with supervision in the overall framework. To this effect, Holloway and colleagues developed a systems approach to supervision (SAS) model whose relevance to this research work was premised on the relationship between the supervisor and supervisee as the main determinants of appropriate supervision [12]. Relatedly, another integrative model developed by Bernard and Goodyear consisting of three phases (intervention, conceptualization and personalization) and three supervisor roles (teacher, counsellor, and consultant) has been widely used in supervision studies [13]. While, these models have been developed in high-income countries where supervision frameworks are well defined and human resources for supervision are adequate, in low income countries such as Uganda, supervision frameworks for private drug sellers are absent and trained human resource for supervision is an ever present challenge [14]. Moreover, both Bernard’s discrimination model and the SAS model are devoid of the peer-to peer element which may be critical in addressing the present human resource challenge as far as supervision of the private sector in low income countries is concerned.

Given the increasing role of drug sellers in providing health care in rural settings and the documented evidence of quality support supervision, the peer supervision model- a type of supervision where people of similar hierarchical status or who perceive themselves as equal encourage and enhance learning and development between each other was proposed as a possible method of supervision [15]. Having proved successful in different settings, it was envisaged that peer supervision would improve treatment of children with pneumonia symptoms, uncomplicated malaria and non-bloody diarrhoea among drug sellers [16–18].

### Aim of the study

This study aimed at constructing a model of an appropriate peer supervisor for private drug sellers at district level in rural Uganda based on views of drug sellers and their inspectors.

#### Drug shop regulation in Uganda

Generally, two types of private pharmacies exist in Uganda. That is, type I and type II. Type I pharmacies are operated by a registered pharmacist and can sell both prescription and over the counter drugs. On the other hand, type II pharmacies commonly referred to as drug shops are the majority of the two types of pharmacies and are mainly found in rural areas serving a population generally not reached by public health facilities. Type II pharmacies are allowed to stock and sell class C drugs. According to the National Drug Authority, Class C drugs include; oral preparations, vitamins and minerals, topical preparations and some anti-microbial formulations [19]. In Uganda, pharmaceutical regulation is a mandate of the Ministry of Health delegated to the National Drug Authority (NDA).

The NDA works together with the District Health Officer (DHO) and District Drug Inspector (DDI) to inspect drug shops at district level. Under the office of the DHO is a committee which includes the DDI, the assistant district health officer in charge of the environment, district health visitor as well as the records officer. According to the guidelines for inspection of drug shops, the person in charge of inspection should be qualified at bare minimum as a pharmacy technician. A pharmacy technician is one who has attained a diploma in pharmacy, has worked under supervision of a pharmacist for at least 1 year and has been trained to dispense drugs. The main regulatory functions of the district drug inspector as stipulated in the recruitment guidelines for health workers in local government include; ensure that essential, safe, efficacious and cost-effective drugs are made available to the entire population, make a continuous review of the needs, knowledge and resources of essential drugs, provide systematic public information and professional training and retraining of health workers. The district drug inspector is also mandated to intensify research in all types of drugs including traditional medicines, ensure compliance of health workers with the international regulations on drugs including the conventions on narcotic drugs and psychotropic substances, inspection of suitability of premises for drug shops, making sure only qualified staff operate in drug shops, tracking illegal drug sellers and ensure that expired drugs are not mixed with usable stock [20]. The DDI is mandated to carry out inspection on a monthly basis. However, there is no law or policy framework guiding the supervision of drug shops in Uganda. The main assumption by law makers is that once a drug seller is qualified as per the statutory requirements for operating a drug shop, they will be able to supervise themselves. This has not been the case as evidenced by the continued inappropriate treatment of febrile children under 5 years by drug sellers [8, 21].

## Methods

### Study setting and participants

This study was conducted in two rural districts namely Luuka and Buyende in East-Central Uganda. It is estimated that both districts will have a combined population of 675,600persons by December 2020 [22]. Personal attributes that could be used to trace the people involved in this study were not disclosed in order to minimize the potential of being identified. A total of six inspectors (four at district and two at national level) were purposively sampled for key informant interviews. Inspectors interviewed at district level included; District Health Officers (DHOs) and District Drug Inspectors (DDIs). On the other hand, licensed drug sellers were purposively chosen for focus group discussions.

### Selection of informants

Purposive sampling was used to recruit licensed drug sellers in the two study districts in East-Central Uganda. Six Key Informants (KIs) who are the statutory designated inspectors were approached by the lead investigator (AB), while 100 and 30 drug sellers comprised of nurses and nursing assistants were invited for focus group discussions by the DDI with permission from the DHO. All the inspectors at national and district level were male with a combined average (standard deviation) age of 47(5.1) years and had been in service for 7.3(2) years. In both districts combined, there were more female (64) than male (49) nursing assistants. However, there were more male (10) than female (7) nurses. Overall, the male drug sellers were older and had spent more years in service than the female drug sellers as shown in Table 1. In both districts, drug sellers had already been trained on how to manage febrile children less than 5 years by the Clinton Health Access Initiative (CHAI)-a non-governmental organisation. The choice of sampling technique was based on the fact that researchers wanted to achieve diversity based on gender, academic qualification and region of operation [23].

**Table 1:** Characteristics of participants

Variable	Cadre		
	Supervisors	Nurses	Nursing assistants
**Gender**			
Male	6	10	49
Female		7	64
**Average age (SD)**			
Male	47 (5.1)	36 (8.3)	37 (8.2)
Female		32 (8.1)	30 (5.8)
**Average years in service (SD)**			
Male	7.3 (2)	8.4 (4.6)	11.5 (6.3)
Female		8.3 (5.7)	6.8 (5.8)

Inspectors stipulated by statutory regulations were selected as key informants for this study as these were the most appropriate cadre to answer the research question [24–26]. In one of the study districts, drug sellers had an association with a duly elected chairperson and leadership structure which was absent in the other district. The drug shop association worked with the district drug inspector to ensure that unlicensed drug sellers registered and got an operating license with ease. In both districts, the DDIs derive their inspection mandate from the DHO. Also, in both districts, drug sellers engage in self-supervision after they have been licensed by NDA as stipulated by national policy guidelines [7].

### Data collection

A discussion guide was developed based on research of how to conduct feasibility studies [27]. FGDs with drug sellers lasted between 50 and 110 min while KII lasted between 45 and 60 min. All interviews were audio recorded and transcribed verbatim using Atlas ti.7 software. Respondents were asked whether they felt peer supervision would be a good method of supervising drug shops, whether it would be embraced as an alternative to self-supervision and whether it could be used to augment inspection. In addition, participants were asked what resources they felt should be given to peer supervisors to make the process successful.

### Data analysis

Collected data was coded and analysed over a 2 months period using a constructivist grounded theory method as laid down by Charmaz [24]. During initial coding, incidents from transcripts of the first two FGDs were coded and compared. The incidents were then collapsed into categories in the initial analysis [25, 28]. Coding was done according to the meaning of the incidents and relevance to the study to form meaningful concepts [24, 28]. The aim of initial coding was to fracture data to make constant incident to incident comparison while observing for any emerging data patterns. Three levels of coding were used in the process; initial, focused and theoretical coding [29]. This process was repeated in an iterative manner with more codes and categories being developed from subsequent interviews if and when they were non-existent from already developed categories. This iterative procedure was also applied during the final stages of analysis using inductive and deductive thinking and reasoning [30].

In the process of code generation, identification and labelling of key words with intention to assign meaning to data occurred. The labelling enabled researchers of the study to make comparisons between the developed and developing codes by way of constant comparison which aided in forming sub-themes and themes. Initial analysis of the developed codes led to theoretical sampling-following leads in the data by sampling new participants who provided relevant information. This was to ensure that any subsequently collected data saturated categories that were being developed necessary for theory development [31]. This was followed by focused coding which identified core categories by building basic data into abstract concepts until theoretical data saturation was achieved [29]. Simultaneously, we increased theoretical sensitivity by re-reading literature about supervision in general and peer supervision in particular. Other ways of improving theoretical sensitivity included initial data coding, category building and reflection through memoiring. Final synthesis of categories derived from coding and analysis was done through theoretical coding in order to create the new context appropriate model for a peer supervisor [32, 33]. Transcripts from the six key informants were transcribed and arising themes infused in construction of the model. Table 2 illustrates the coding process.

**Table 2:** The coding process

Open codes	Focused codes	Theoretical codes
Check diagnosis, treatment, check education level, room space, storage space, license,should work with DDI, should work with parish co-coordinators, should work with sub-county chairperson, have powers to suspend, respect others, not ask for money, should carry ID	• Instructing and monitoring • Organisation structure • Professional ethics and standards	Clearly defined roles
Peer will teach you new things, may not supervise competing drug sellers, challenge advice, eliminate laziness, educate through workshops, deal away with segregation, selection by peers, selection by ballot papers, selection by raising hands, organise training, workshops, training every three months	• Case conceptualization • Cultural world view • Learning goals and styles • Participatory peer selection • Theoretical orientation	Role model
Peer given cash right away, remuneration range between one to ten thousand shillings, remuneration based on distance moved, pay via mobile money, appreciation not bribes, needs allowance, transport for supervision, needs bicycle, needs motorcycle, transport refund	• Remuneration • Transport	Incentives
Stops drug sellers from fleeing, averts fear, averts fraudsters, reduces harshness, treats us in a more friendly manner, phone call precedes visit, people person, advice precedes punishment	• Desensitisation • Interpersonal style • Counseling skill	Mediation

## Results

**Fig. 1:**
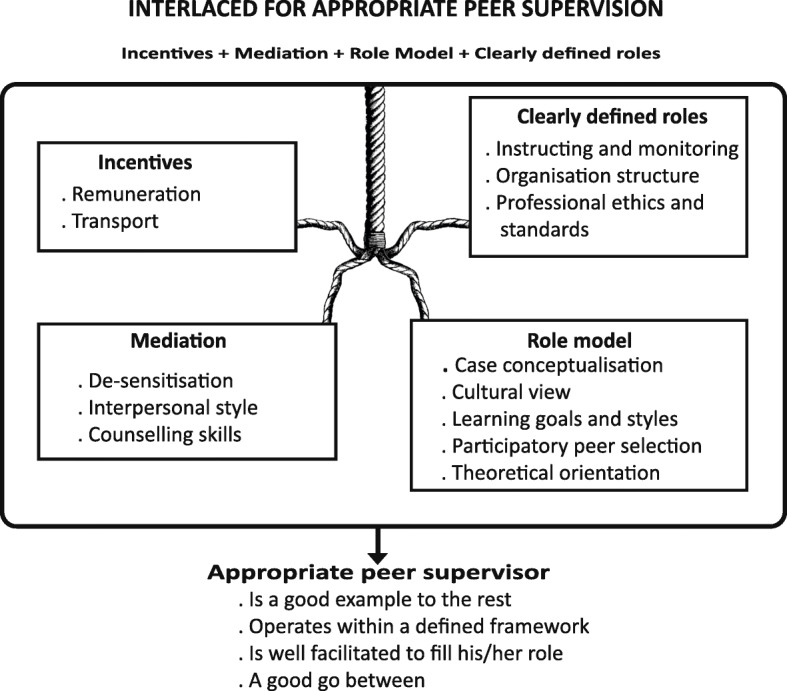
Model of an appropriate peer supervisor

Construction of the final appropriate peer supervision model involved iterative processes which included analysis and synthesis of raw data. This overarching appropriate peer supervision model is accentuated by four sub-dimensions namely a) Incentives b) clearly defined roles c) mediation d) role model. The different sizes of the developed dimensions arising as a result of the different components therein highlight the varying time and resources that will be required for each dimension to accentuate appropriate peer supervision. The biggest dimension will require the most time and resources (Fig. 1). Nevertheless, all dimensions will synergise one another in an iterative manner with no single dimension being sufficient on its own but rather all four working in tandem to define an appropriate peer supervisor. Figure 1 is the constructed model for an appropriate peer supervisor for private drug sellers at district level in rural Uganda.

### Role model

Role modelling seemed to be the most talked about dimension of peer supervision. Drug sellers said that a good peer supervisor was one who was more educated than the rest and as such, will be able to conceptualise complicated cases through his or her professional experience and give advice. The drug sellers preferred that advice be passed on in form of counselling rather than the traditional harshness associated with government inspectors. Relatedly, the drug sellers felt that since peer supervisors would be chosen from among them, they would understand challenges faced and therefore be a little more lenient during supervision which would yield better outcomes than locking up drug shops as has been during inspection visits.

*“You can even tell them the challenges you encounter during your operations and they can advise you on what to do or help you make corrections where possible” (P20: FGD5, comprehensive nurses, district without drug shop association)*

As far as the inspectors were concerned, there was a general perception that if there was a way of identifying a role model from amongst drug sellers who would mobilise fellow drug sellers, this would ease the licensing process. Drug inspectors become harsh when they find unlicensed drug sellers prescribing and dispensing drugs. In the event that it was not possible to choose from the existing drug sellers, the inspectors suggested choosing people from the district local government structure. The inspectors suggested having sub-county and parish mobilisers in the event that it was not feasible to have appropriate peer supervisors selected from drug sellers.

*“Possibly, if we can come up with strategies of identifying people from amongst them [drug sellers] to act as mobilisers, that can encourage them to license and bring them to us so that we have a free atmosphere. We have sub county structures. Parish mobilisers can play a big role if we work together to improve the services of drug sellers.” (KII, district without drug shop association)*

Confidentiality was another attribute drug sellers felt was very important for one to be considered a good peer supervisor. This is because besides providing clinical services, the goal of selling drugs is to make profit. This introduces an element of competition among fellow drug sellers. The drug sellers mentioned that they preferred a peer supervisor who maintained the highest level of confidentiality and was not the kind to divulge too many personal details. The drug sellers felt that if the peer supervisor was to disclose too many business secrets, this would jeopardise the trade resulting in clients preferring some drug sellers over the others. Some issues raised by drug sellers included misdiagnosing patients and offering faulty treatment.

*“Also I might not supervise the colleague whom we don’t get along with because he takes my clients away” (P24: FGD3, nursing assistants, district without drug shop association)*

In a bid to identify a peer supervisor who maintains confidentiality, drug sellers said they were very comfortable identifying such a person themselves through a transparent and democratic process. They felt that they knew each other and that they knew who was and who was not capable of maintaining confidentiality and supervising appropriately. This democratic process would also ensure that the system does not impose a tyrant on them who would make the running of business very hard. When asked how they preferred to choose the peer supervisor, some drug sellers preferred the ballot method while others preferred either raising their hands in support or lining at the back of the preferred candidate. Drug sellers also said that they preferred peer supervisors going for further training facilitated by the government and receiving a monthly allowance if time and opportunity allowed.

*“I think on the issue of the peer we should be choosing that person ourselves. Secondly; if there is any opportunity such as an organization or the government providing them with some money, let them go and study and be at higher level than us concerning establishment of a drug shop. Let them also be given an opportunity of receiving salary every month after all, they will be working amongst us” (P16: FGD1, nursing assistants, district without drug shop association)*

Drug sellers also felt that besides adhering to high standards of confidentiality, a good peer supervisor was one who will have a balanced cultural world view. In the course of our verbal interaction with the drug sellers, they mentioned that conflict amongst them was natural and it would be good if they got a peer supervisor who would not take any sides if conflict arose. They mentioned that conflict arises from relationships as well as through business competition. They felt that it would be good if the peer supervisor remained neutral and stuck to his supervisory role and did not interfere with other social problems.

*“Another view madam is that I may not be getting along well with another drug seller especially my neighbour because we might be sharing the same woman. Or, the patients prefer my medication to his. So, the peer supervisor should deal away with segregation.” (P22: FGD3, nursing assistants, district without drug shop association)*

Drug sellers also felt that an appropriate peer supervisor is one who would advocate for more workshops and seminars which would help everyone get more knowledge about common childhood illnesses thus improving treatment through advanced learning. They also said that a good peer supervisor would be one who promotes hard work in business since that supervisor would do their job more routinely.

*“We talked of workshops and seminars for those peer supervisors but even we the drug sellers also need to be trained in such seminars such that we are updated of any new developments.”*

*(P15: FGD4, nursing assistants, district with drug shop association)*

### Clearly defined roles

This category was talked about with clarity by drug sellers because of the experience they had with government inspection which has many actors and is largely unstructured. To the drug sellers, an appropriate peer supervisor is one who will have predictable timing and routine of supervision. Drug sellers also felt that a good peer supervisor is one who will have appropriate supervision tools. Such tools will include a supervision check list. The drug sellers said that peer supervisors will need to have clearly defined tasks such as checking on education levels of drug sellers, operating room space, storage area for drugs, presence of toilet and hand washing facilities. Not being so intrusive to the extent of reaching drug sellers’ bedrooms in search for illicit drugs was another concern that was raised. In all, they mentioned that a peer supervisor would be a good first line supervisor before other layers of supervision take precedence if they exhibited professional ethics and standards.

*“Some have a policy of coming up to where we sleep in search of drugs and for me I think that has to change. They should know their boundaries and only work within those limits. Just in case they find anything wrong within those limits, then I can seek for an apology.” (P15: FGD4, nursing assistants, district with drug shop association)*

In having clearly defined roles, drug inspectors also felt that peer supervisors ought to work within a clearly defined organisational structure. This was stressed by one key informant as he said that when drug sellers are under one organisation, they are easy to regulate and following them up is easy. He intimated that the original thinking behind initiation of drug shops in the country was to act as a temporary stop gap measure for government inadequacies. However, the key informant was dismayed at how drug sellers through their organisations had become so powerful to the extent that they had dragged the NDA to courts of law. He said that every attempt at trying to streamline drug sellers is treated with a lot of suspicion. He lamented that the drug sellers had gone to great lengths to undermine policy by forming associations even where they were non-existent previously.

*“I think it is a good initiative. Because in our systems, these [drug sellers] were supposed to be temporary stop gaps but I can see they are entrenched. They are down there serving the poor and hard to reach. You have heard of how they [government] tried to faze them out but you have heard the noise they have made. I think even those who had no association have organized them.”* (*KII, inspector, NDA*)

### Incentives

This dimension comprised three properties namely transport, remuneration and a combination of transport and remuneration. Drug sellers and inspectors recognised the need to offer appropriate incentives if the supervision process was to be smooth. While some drug sellers felt that either giving money in form of a monthly stipend or a bicycle was enough for smooth peer supervision, others felt that an amalgamation of the monthly stipend and a bicycle were the most appropriate incentives for time spent during supervision. The drug sellers suggested that government should buy the bicycles and the money should be given as a transport refund.

*“They should be given at least a bicycle and allowance to facilitate their supervision.” (P24: FGD3, nursing assistants, district without drug shop association)*

On the other hand, inspectors also felt that putting in place an incentive was a good initiative for peer supervision. The inspectors said that the incentives would best be executed at the level of drug shop associations if well managed. This would benefit both the drug sellers and the peer supervisors. They emphasized the fact that the incentives should be passed on through the drug shop association because the association was formed at district level. As such, the drug shop association would be controlled by district authorities and not be a parallel structure.

*“May be at district level, these associations would work when they are better organized. Some of them were saying these people are just getting money. But I think if they are well organized and people know the benefits [of incentives], they can work out.”* (*KII, inspector, NDA*)

### Mediation

The dimension of mediation was divided into three namely: de-sensitisation, interpersonal style and counselling skills.

Drug sellers in the focus group discussions felt that an effective peer-supervisor was one who would be able to diminish negative tendencies associated with fear of government inspectors in what was termed as de-sensitisation. This is because during inspection, government inspectors ensure that errant drug sellers are arrested and their drugs are confiscated. In addition, they are insulted and ashamed in front of patients. Whereas this is more common amongst the unregistered drug sellers, the registered drug sellers tend to take no chances when government inspection is on-going. For this reason, participants in the focus group discussions felt that they would prefer someone who will be recognised as a first line supervisor before the more superior supervisors intervene. These assertions are highlighted in the quote below.

*“I know those people [peer supervisor] will be approachable and not mistreat us. What they will do is to make a report and tell us where we have done well. They will not be as harsh as those people [inspectors]. That is why those people [peer supervisors] should be chosen from amongst us.” (P17: FGD 2, Nursing assistants, district without drug shop association)*

As far as interpersonal style of the peer supervisor was concerned, participants in the focus group discussions said that since their businesses were running on very little capital, they would prefer someone who understands the challenges of raising such capital and the losses incurred when drugs are confiscated. As such, for one to be considered an appropriate peer supervisor, that person will need to be sociable, of good character and able to help when there is an overwhelming number of patients. The following quote captures what was said.

*“That’s why we mentioned that they should train the peer supervisor to have social manners and be able to assist when they get to your drug shop and find many clients.” (P28: FGD2, nursing assistants, district with drug shop association)*

Having good counselling skills was another attribute mentioned for one to be considered an appropriate peer supervisor by drug sellers in the focus group discussions. The drug sellers told the lead investigator of the study (AB) that in the event they were caught doing the wrong thing, they preferred being advised and warned before being punished as is the norm with government inspectors.

*“For me I think that if they [peer supervisors] get me with something am not supposed to do, say a drug, they have to first warn me and if I repeat the same thing again, they can report me” (P15: FGD4, nursing assistants, district with drug shop association)*

## Discussion

This study aimed at constructing a model of an appropriate peer supervisor for private drug sellers at district level in rural Uganda based on views of drug sellers and their inspectors. Effort was put into understanding how the model fitted within the already existing models of supervision. This aim was against a plethora of published evidence on the continued inappropriate treatment of paediatric febrile illnesses associated with the existing method of self- supervision in Uganda. Findings reveal a complex nexus of individual, institutional and policy challenges augmented by the fact that there is no clear framework under which public resources can be allocated for supervision of the private sector. The dimensions that emerged from theory building interlaced for appropriate supervision. There is need for government to pilot peer supervision among rural drug sellers to purge the existing supervision gap. Themes from the data are discussed in the section below.

### Comparison with Bernard’s discrimination and the SAS models

As depicted in Fig. 1, role modelling seemed to be the most talked about dimension of peer supervision. Drug sellers preferred someone they could easily relate with given the context in which they operate. This relationship has been found to be pivotal and in agreement with both the SAS and Bernard’s discrimination models as precursors for appropriate supervision [11]. However, Bernard’s discrimination model does not elaborate in detail the supervision relationship which makes it hard to compare with results of this study. There is growing support for the need to improve supervisor-supervisee relationships because this improves internal support supervision quality rather than supervision frequency which is emphasized by many government agencies involved in supervision at unit level like drug shops in low income countries [14, 34]. As far as rural drug sellers are concerned, this can best be achieved when the supervision relationship between the supervisor and drug seller is cordial.

In addition, drug sellers wanted clearly defined roles of supervisors and a defined list of expectations for drug sellers. This is based on the current situation where for instance, inspectors have certain expectations from drug sellers which are largely prescribed by the law and do not expect drug sellers to have any expectations. This notion of expectations being uni-lateral is not in agreement with Bernard’s discrimination model which is more prescriptive when it comes to defining roles. In Bernard’s model, supervisors adjust according to the needs of the supervisees. Hence, the supervision style for novices is different from expert supervisees.

In our study however, we could not apply Bernard’s discrimination model because drug sellers from the two districts had already been trained on how to manage febrile children less than 5 years by the Clinton Health Access Initiative (CHAI) and were assumed to be at the same level in terms of appropriate treatment. More applicable was the SAS model where drug sellers wanted the roles of the peer supervisor to be less explicit and not to exceed formative and summative evaluation functions. However, while using the SAS model, caution should be exercised because the common assumption is that once a drug shop has been licensed, the seller operating the drug shop should engage in the right practise. This notion of self-supervision with no superior and authoritative level of supervision obliterates feedback which is the whole mark of supervision. Research shows that when supervision is structured, the process offers an opportunity for feedback, self-assessment, and peer assessment [35, 36]. This can only happen when there are clear terms of engagement handed to supervisors by the organisation responsible for supervision as well as a clear definition of who does supervision, how and when it occurs [37, 38]. From our study, there was no structure responsible for supervision of drug shops other than relegating the supervision function to the duly licensed drug sellers. This deviates from what the SAS model prescribes and makes the whole self-supervision process untrustworthy, unprofessional and prone to abuse.

Furthermore, that which drug sellers referred to as the government approach to supervision was actually inspection and was referred to as a financial burden transferred to drug sellers. This is because drug sellers felt that the fuel refund demanded by DDIs every time they made an inspection visit to drug shops was unfair since the drug sellers pay annual license fees and other statutory taxes. Moreover, the government facilitates DDIs on a monthly basis to carry out their inspection mandate. This dimension of incentives is not talked about either by the SAS or Bernard’s discrimination model hence an extension to the existing models.

In sum, an appropriate peer supervisor was described as one who had the ability to rescind harsh government policies aimed at affecting the day-to-day running of drug shops. Such decisions may include but are not limited to harassment, embarrassment and intimidation of drug sellers. This revelation was made based on the fact that inspection of drug shops in Uganda is carried out in a harsh manner. Since the current method of self-supervision has not resulted in desired treatment outcomes especially for febrile children aged 5 years or less, it is envisaged that democratically selecting peer supervisors with good mediation skills will purge this glaring gap of supervision. This will be possible when the peer supervisors have good counselling skills described and supported by both the SAS and Bernard’s discrimination model [11].

The basic assumption is that such a person would be an influential person trusted by government agencies and drug sellers. This person would be an ideal and efficient first line supervisor before other supervisors at a much senior level get involved. Decisions made by the peer supervisor should make sense to government agencies and drug sellers creating a good environment for offering services to the community while making profit from their business at the same time.

### Policy and program implications

Given the critical service delivery gap filled by drug sellers in underserved areas in many low income countries, this research highlights a critical policy and program area that needs to be addressed. This research demonstrates that inspection in its current state can only ensure compliance with set guidelines albeit with some degree of coercion. However, evidence shows that there is a great deal of uncertainty as to whether inspection alone can improve quality of care among drug sellers in low and middle income countries [39, 40] and yet supervision has been found to be effective in improving the quality of health care [1, 41]. This research therefore points to the fact that there is need to have a supervision policy put in place and a framework under which resources for supervision of private sector drug sellers can be dispensed. It is envisaged that once inspection is augmented with supervision, the appropriateness of treating febrile children will improve.

The research findings show that currently, government inspection which is the closest in terms of supervision is detached from the experience of drug sellers and as such, there is a strained relationship between government inspectors and drug sellers. The inspection process is characterised by fault finding and is aimed at arresting and apprehending offenders rather than counselling with intent to improve practise. Moreover, evidence shows that supervisors are regarded as pivotal by supervisees [42, 43]. This happens most especially when information from supervisees needs to be synthesized and passed on to top level management in a manner that sustains a favourable operational climate. The manner in which the information is passed on must favour both the supervisees and the organisation under which they operate. It is therefore important that under the supervision framework, suitable personnel who are referred to as role models in this study be vetted before they are appointed as supervisors by the responsible government agencies.

Relatedly, it is important that the vetted and appointed personnel be adequately motivated by being provided the right means of transport and sufficient financial resources to carry out supervision. As other studies have shown, it is important to have a good incentive structure clearly communicated by responsible authorities or organisations mandated with supervision [44]. This enables supervisors not to be passive, absent or adopt unwanted behaviour such as soliciting illicit funds from drug sellers [45, 46]. It is well understood that financial resources are very scarce especially in low and middle income countries. However, with an appropriate supervision framework in place, the cost of supervision can be cost shared by the private sector, government and development partners where possible. This would go a long way in improving the quality of care by drug sellers for febrile children less than 5 years of age. Introduction of a supervision framework will also go a long way in reducing harshness through mediation while improving the quality of care as has been mentioned in studies done elsewhere [1, 47].

### Areas of future research

Views gathered from this study were used to construct a model for an appropriate peer supervisor. It is important that this model be implemented to test how good it is in the context of rural drug sellers. During implementation, evidence gathered will reveal whether all four dimensions of the model are necessary for the model to be effective or some dimensions can be done without. Field implementation of the model in the rural area will be very important since almost 60% of the people in Sub-Saharan Africa live in rural areas [48].

It is also important that future studies consider how cost effective the peer supervision model is and what other adjustments can be done to this model to make it affordable for drug sellers. It is important that the peer supervision model is cost effective otherwise, the drug sellers pass on this high cost to the end users who are caregivers of febrile children. In addition, it is important to know whether once implemented, the peer supervision model will be embraced by drug sellers since the model involves people engaged in the same trade who may be competing with each other already.

### Study limitations

This study did not use therapy quality scales (TQS) to measure general and specific skills of inspection and peer supervision during data collection [49]. Instead, views from participants were explored using a constructivist grounded theory approach by Kathy Charmaz [24].

Therefore the accounts on peer supervision are characterized by subtle meaning of participants’ perceptions and should not be interpreted as actual measurements of appropriate peer supervision. Although we present findings from drug inspectors, strictly speaking, inspectors are mandated to uphold the law by looking out for errant drug sellers. In essence, we interviewed them because there was no other authority charged with supervising private drug sellers. As such, our work has several areas of concordance and deviance typical of exploratory qualitative studies [50].

The drug sellers interviewed in this study were duly licensed and trained by CHAI. Results gathered from this study can therefore be generalised to other drug sellers in low income countries that have been registered and or licensed in accordance with statutory laws and received some form of medical training. The researchers were cognisant of the fact that unlicensed drug sellers also exist and provide a service to communities in which they live. However, because they work illegally and stand a high chance of being prosecuted when found, it is highly unlikely that the views expressed by drug sellers in this study would apply to the unlicensed drug sellers. This may have created a bias because not all drug sellers that serve the community were included in the study and yet their views may have been different and vital in enriching the study results.

## Conclusions

Four central contributions advance literature by the model developed by our study. First, the model fills a supervision gap for rural private drug sellers. Second, it highlights the need for terms of reference for peer supervisors. Third, it describes who an appropriate peer supervisor should be. Lastly, it elucidates the kind of resources needed for peer supervision.

## Data Availability

Datasets used during the study are available from the corresponding author on reasonable request.

## Abbreviations

CHAI: Clinton Health Access Initiative
DDI: District drug inspector
DHO: District Health Officer
FGD: Focus group discussion
KII: Key informant interview
NDA: National Drug Authority
SAS: Systems approach to supervision
TQS: Therapy quality scale
